# Clinical epidemiology and molecular analysis of hospitalized children with hand-foot and mouth disease during 2009 in Shanghai

**DOI:** 10.1186/1753-6561-5-S6-P46

**Published:** 2011-06-29

**Authors:** H Yu, FX Yan

**Affiliations:** 1 Department of Infectious Diseases, Children's Hospital of Fudan University, Shanghai, China

## Introduction / objectives

We retrospectively analyzed the clinical features and epidemiology of 1386 children with hand-foot and mouth disease during 2009 at Children’s Hospital of Fudan University and investigated some risk factors with fatal cases. Besides, we also identified the pathogen of 116 patients.

## Methods

All the clinical records and laboratory results were collected. A retrospective study was performed and reverse transcriptase-polymerase chain reaction (PCR) assay was used to identify the pathogen.

## Results

A total of 1386 patients were enrolled in this report, with onset median age 25 months. Among them, 62.4% patients aged between 1 to 3 years and 67% patients came from rural area. Fever (88.3%), rashes (99.2%), cough (22.5%), vomiting (25.4%) were the most frequent symptoms while myoclonus jerk, hypertension and tachycardia mainly occurred in those fatal cases. Fatal patients had higher fever, white-blood-cell counts and blood glucose compared to those in stage 1and 2(P<0.05), but not in C-reaction protein or cerebrospinal fluid white-blood-cell counts. Besides these, we also made etiologic analysis of 116 patients to identify 76 cases of enterovirus 71 infection, 4 cases of CA16 infection and 8 cases of other enterovirus infection. And we found the Shanghai EV71 belonged to subgenotype C4 by the phylogenetic analysis.

## Conclusion

The children under 3 years especially from rural area are susceptible to the HFMD infection. Persistent high fever and frequent vomiting, or myoclonus jerk, hypertension and tachycardiamay indicate severe tendency. Some laboratory examinations can help us find the fatal cases earlier. But a early pathogen identification especially enterovirus 71 is so important.

## Disclosure of interest

None declared.

